# Supplementary material for: Design of high-affinity binders to immune modulating receptors for cancer immunotherapy
Source: Nat Commun. 2025 Feb 26;16:2001. doi: 10.1038/s41467-025-57192-z (PMC11865580; doi:10.1038/s41467-025-57192-z)
Supplement: Supplementary file 1 — Supplementary Information [file 41467_2025_57192_MOESM1_ESM.pdf]

Supplementary Information

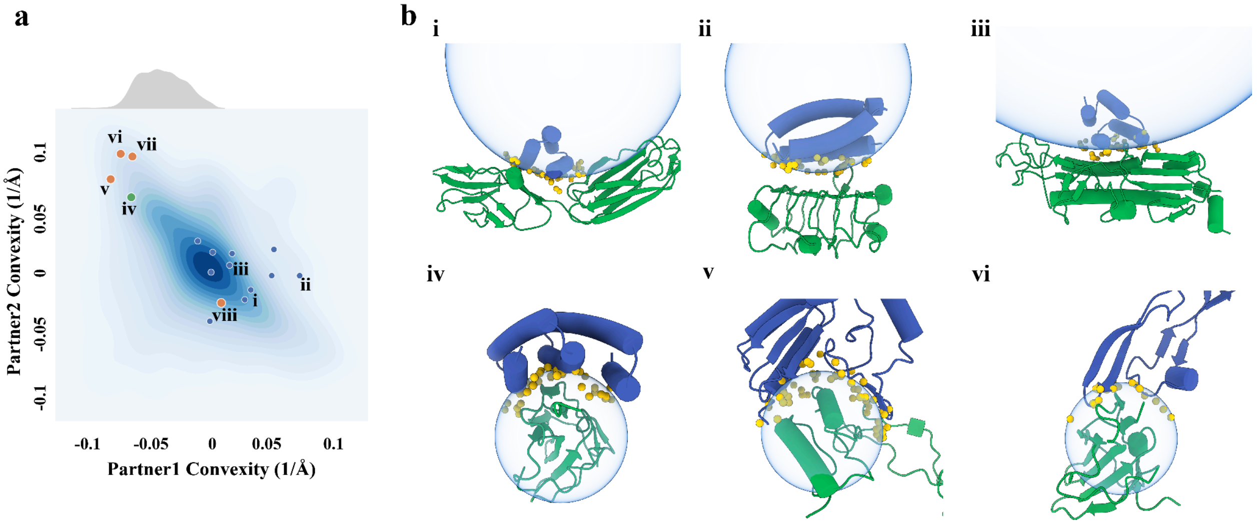

Figure 1. Distribution of protein-protein interface curvatures and examples of concave and convex surfaces. a, Distribution of protein-protein interface curvatures from the PDB and designed protein binders. Blue dots: previously designed protein binders (for these, the designed binders are partner 1 and the targets, partner 2). Previously designed protein binders[2] have been limited to binding to flat or concave interfaces (receptor convexity <=0). Orange dots: examples of native protein complexes, v: PDB ID, 5XXB; vi: TGFβIII/TGFβRII complex, PDB ID, 1KTZ, vii: CD86/CTLA-4 complex, PDB ID, 1I85, viii, PD-1/PD-L1,PDB ID, 3bik. The TGFβRII and CTLA-4 functional interfaces showed high convexity, which we used as case studies to design concave binders. Green dot: The 5HCS scaffolds described in this paper can target convex binding sites. The distribution of convexity of the 5HCS scaffolds (upper part of panel a) shows that the 5HCS scaffolds are diverse enough to cover most of the naturally existing convex interfaces. **b.** Design models of complexes highlighted in panel a. i,ii,ii are PDGFR, 1GF1R, H3 in complex with corresponding *de novo* minibinders; iv, 5HCS binder in complex with TGFβRII; v, PDB ID: 5XXB; vi, TGFβIII/TGFβRII complex, PDB ID:1KTZ. Binders and receptors are shown as blue and green cartoons, respectively. Interfacial heavy atoms from binders are shown as yellow solid spheres. Fitted spherical surfaces are shown as blue transparent spheres.

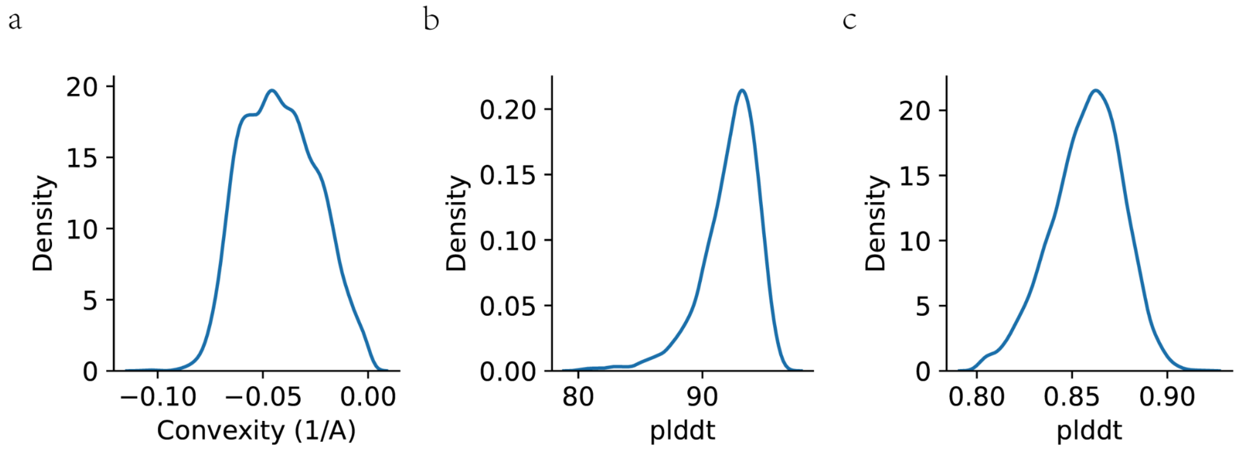

Figure 2. Computational Characterization of 5HCS scaffolds library. a, Distribution of curvatures of the 5HCS library. **b,** Distribution of pLDDT predicted by Alphafold2[1]. **c,** Distribution of plDDT predicted by DeepAccNet[2].

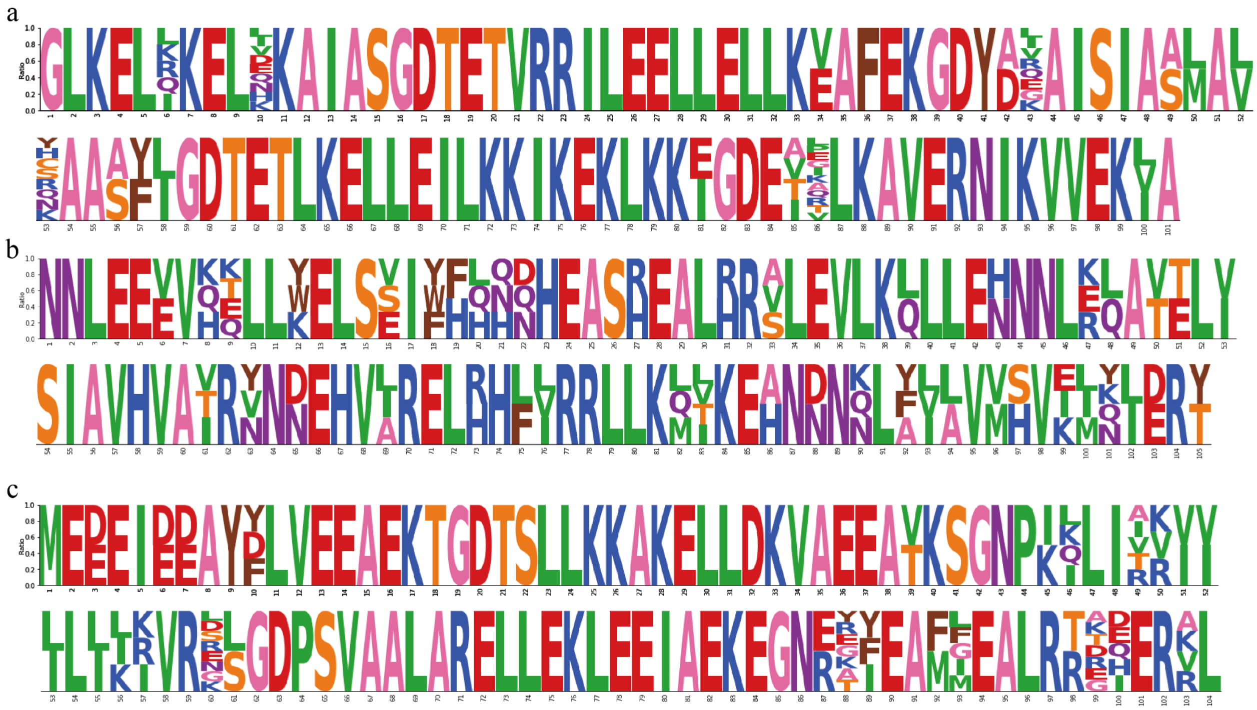

Figure 3. Sequence composition of the combinatorial libraries for affinity optimization. a. Sequence composition of the combinatorial libraries of TGFβRII binders based on 5HCS_TGFBR2_0 sequence. **b.** Sequence composition of the combinatorial libraries of CTLA-4 binders based on 5HCS_CTLA4_0 sequence. **c.** Sequence composition of the combinatorial libraries of PD-L1 binders based on 5HCS_PDL1_0 sequence.

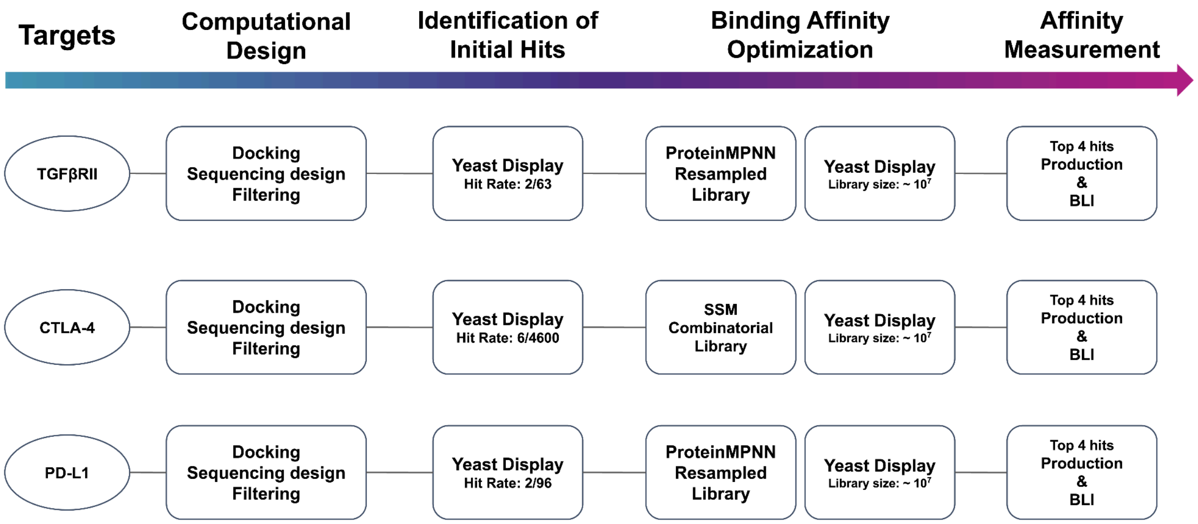

Figure 4. Workflow of Design and Optimization of the 5HCS binders. There are generally four stages for each target including, computational design, identification of initial hits by yeast display (first round of yeast display), binding affinity optimization (second round of yeast display) and final affinity measurement. Library size and hit rates are labeled in the corresponding boxes.

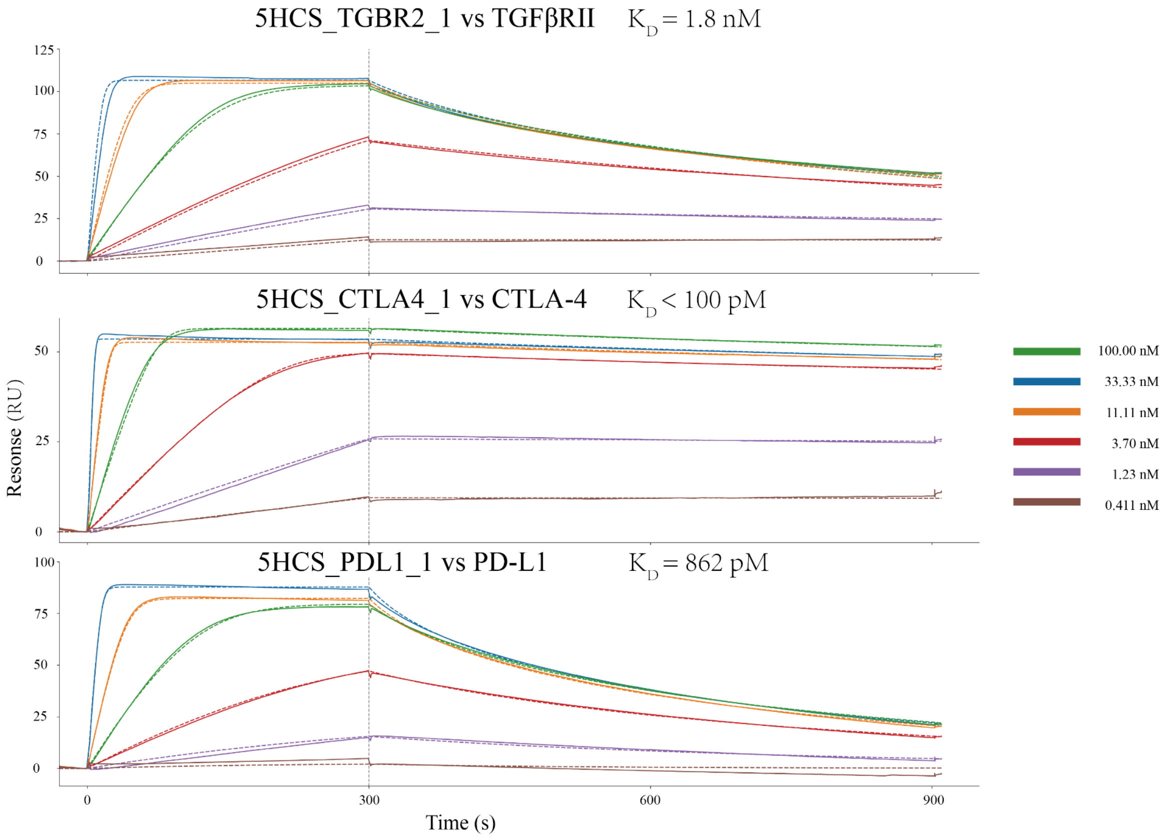

Figure 5. Binding of 5HCS binders to corresponding targets. Binding of 5HCS binder to corresponding targets were measured by Biacore 8K using HEPES running buffer with P20 (0.01 M HEPES pH 7.4, 0.15 M NaCl, 3 mM EDTA, 0.005% v/v Surfactant P20). Target proteins were immobilized on chips(biotin capture chip:TGFβRII, CTLA-4; protein A chip: PD-L1) Binding traces (solid lines) are fitted (dased lines) using Langumuir 1:1 interaction model for kinetic evaluation.

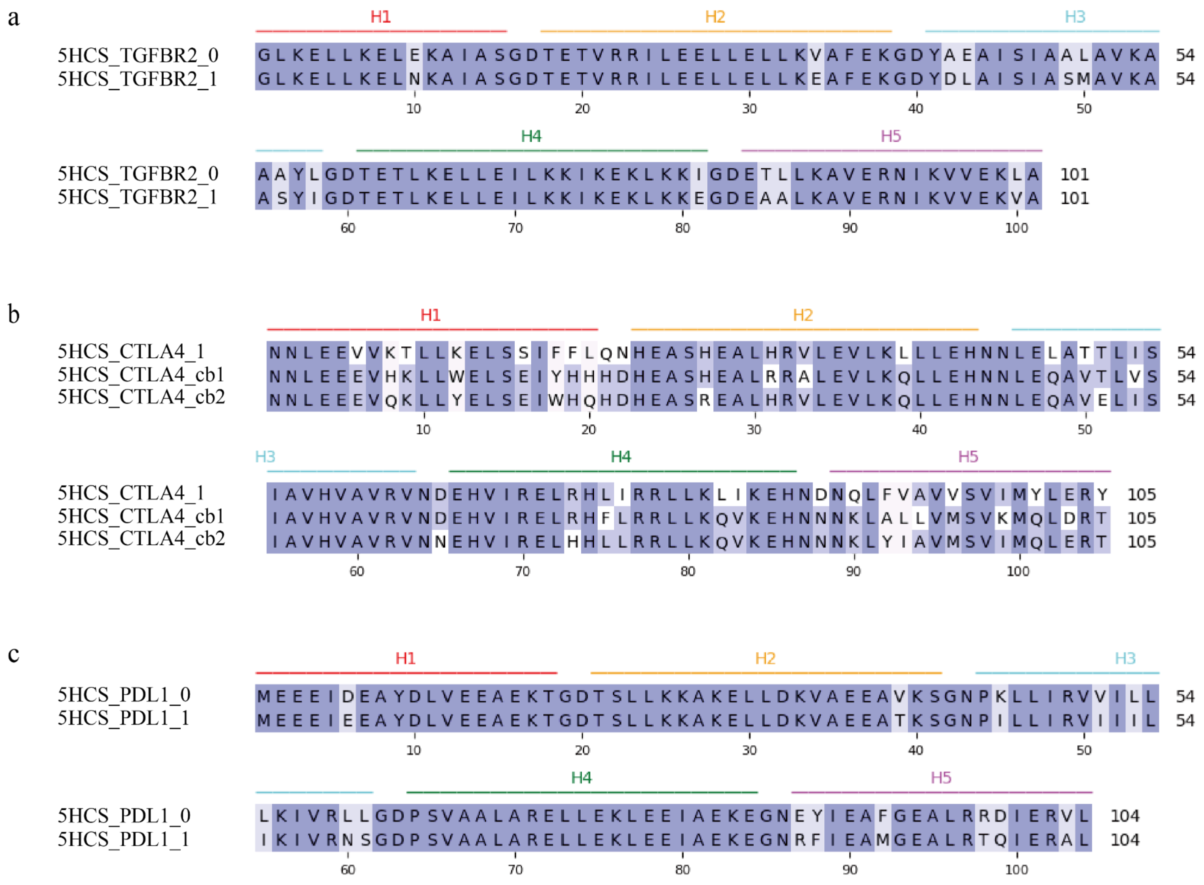

Figure 6. Sequence alignment of initial identified 5HCS binders and optimized binders using combinatorial library. a. Sequence alignment of 5HCS_TGFBR2_0 and 5HCS_TGFBR2_1. The sequence identity and similarity are 88.12 % and 92.08 %. **b.** Sequence alignment of 5HCS_CTLA_0, 5HCS_CTLA_1, 5HCS_CTLA_2. The average sequence identity and similarity between are the initial binder, 5HCS_CTLA_1 and optimized binder 5HCS_CTLA_cb1 and 5HCS_CTLA_cb2 are 72.86 % and 83.34 %. **c.** Sequence alignment of HCS_PDL1_0 and HCS_PDL1_1. The sequence identity and similarity are 86.54 % and 93.27 %.

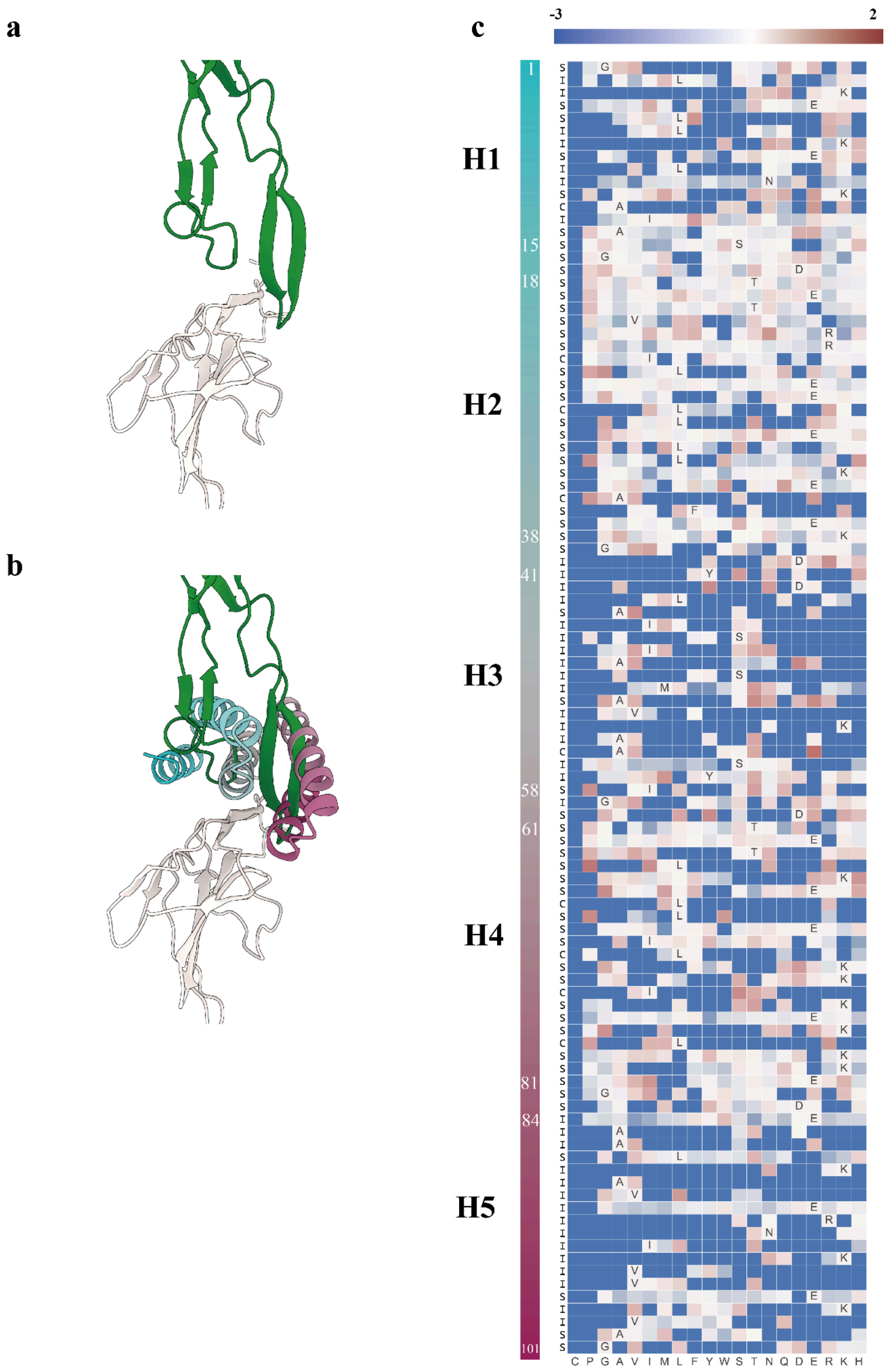
a
b
c
-3
2
H1
H2
H3
H4
H5
1
15
18
38
41
58
61
81
84
101
C P G A V I M L F Y W S T N Q D E R K H

Figure 7. TGFβRII binding protein binding site and SSM analysis. a. Complex structure of the TGFβRII (gray) and the TGFβ-3 (green). **b.** Alignment of 5HCS_TGFBR2_1 (rainbow) design model to TGFβRII (gray) and binds to the TGFβ-3 (green) binding site. **c.** Heat map representing the log enrichments[3] for the 5HCS_TGFBR2_1 SSM library selected with 1.6 nM TGFβRII. Enriched mutations are shown in red and depleted in blue. The annotated amino acid in each row on the heatmap indicates the residue from the parent sequence. Residues are categorized into interface(I), core(c) and surface(S) by their position in the model. The category is listed beside the heatmap on the left.

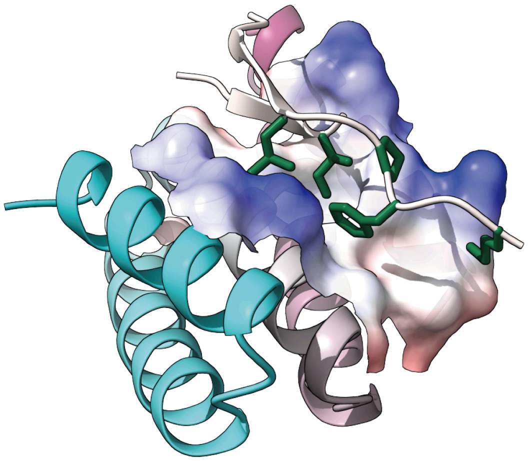

Figure 8. De novo designed binding groove on 5HCS_TGFBR2_1 . 5HCS_TGFBR2_1 shown in cartoon (rainbow) and electrostatic potential surface. TGFβRII binding motif shown as white cartoon and green sticks.

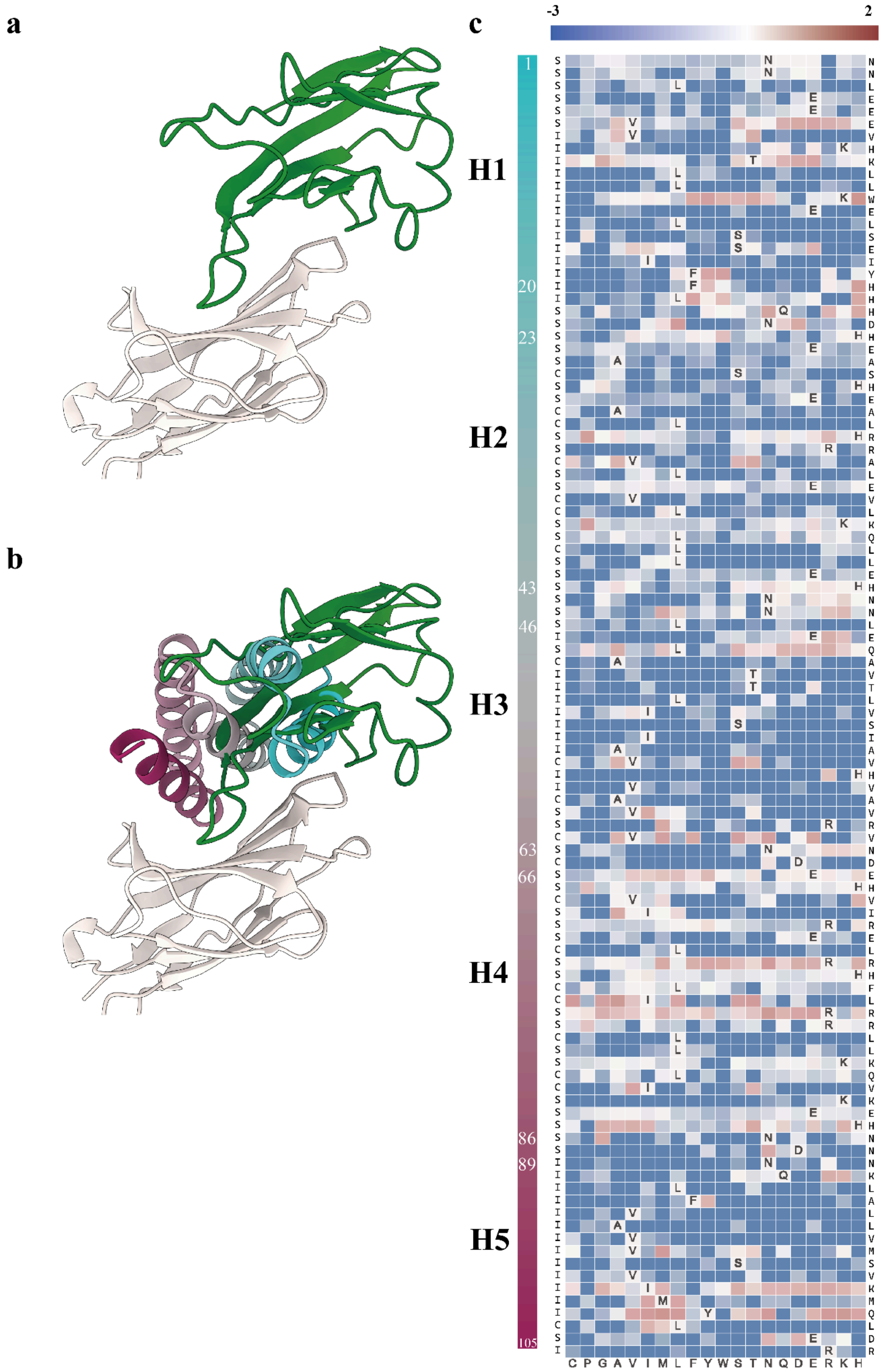

a
b
c
-3
2
H1
H2
H3
H4
H5
1
20
23
43
46
63
66
86
89
105
C P G A V I M L F Y W S T N Q D E R K H

Figure 9. CTLA-4 binding protein binding site and SSM analysis. a. Complex structure of the CTLA-4 (gray) and the CD86 (green). **b.** Alignment of 5HCS_CTLA4_0 (rainbow) design model to CTLA-4 (gray) and binds to the CD86 (green) binding site. **c.** Heat map representing the log enrichments[3] for the 5HCS_CTLA4_1 SSM library selected with 10 nM CTLA-4. Enriched mutations are shown in red and depleted in blue. The annotated amino acid in each row on the heatmap indicates the residue from the parent sequence. Residues are categorized into interface(I), core(c) and surface(S) by their position in the model. The category is listed beside the heatmap on the left. The sequence of 5HCS_CTLA4_1 is listed beside the heatmap on the right.

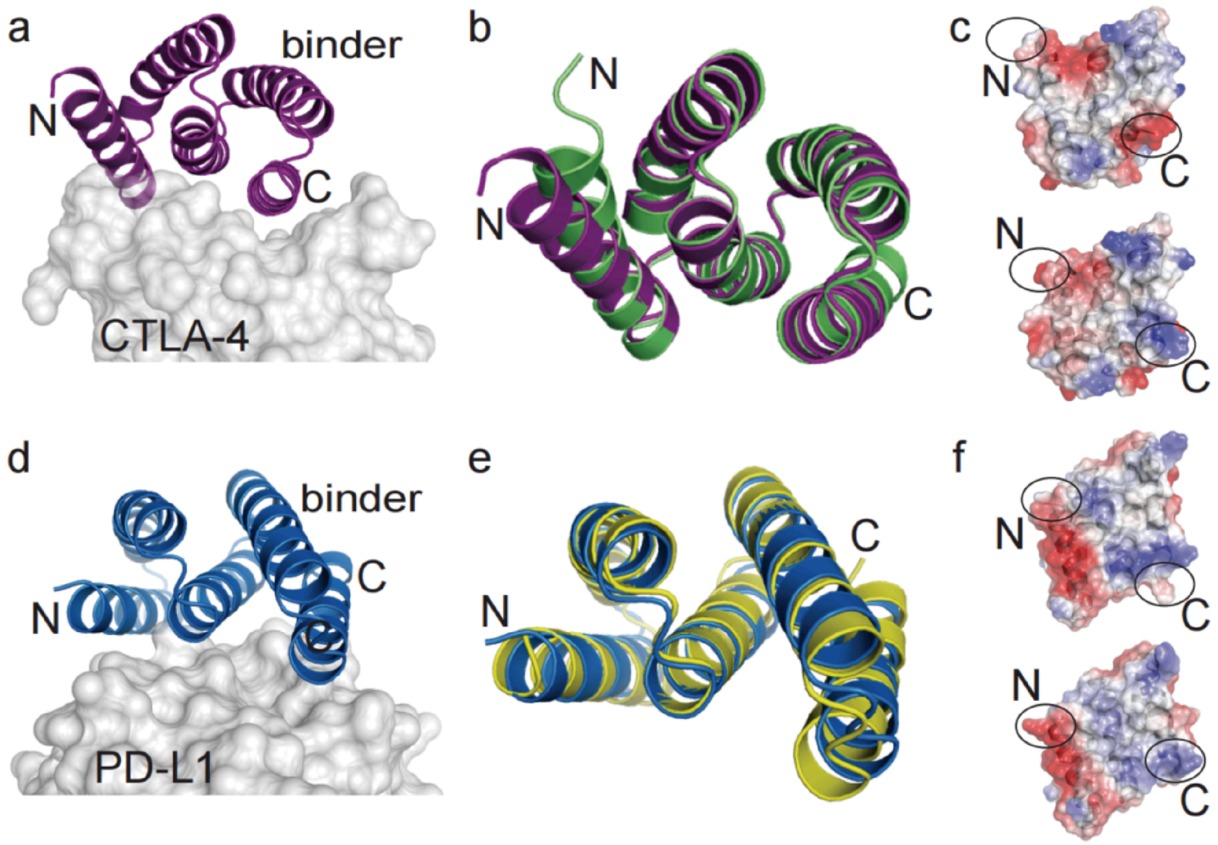

Figure 10. High-resolution structures of 5HCS_CTLA4_2 and 5HCS_PDL1_1.

a, A view of the structure of 5HCS_CTLA4_2 shown in ribbon representation (colored purple). N and C denote the location of the N and C termini. Transparent molecular surface (light gray) depicts modeled CTLA-4 to highlight the binding interface. **b**. Overlay of 5HCS_CTLA4_2 structure to the design model (green). **c**. Comparison of charge distribution at the binder interface between the crystal structure (top) and the model (bottom). **d**. A view of the structure of 5HCS_PDL1_1 (blue, ribbon representation). Part of the modeled PD-L1 is shown as a transparent molecular surface (light gray) **e.** Superimposition of 5HCS_PDL1_1 structure to the design model (yellow). **f.** Electrostatic potential at 5HCS_PDL1_1 interface (top, crystal structure; bottom, de novo model).

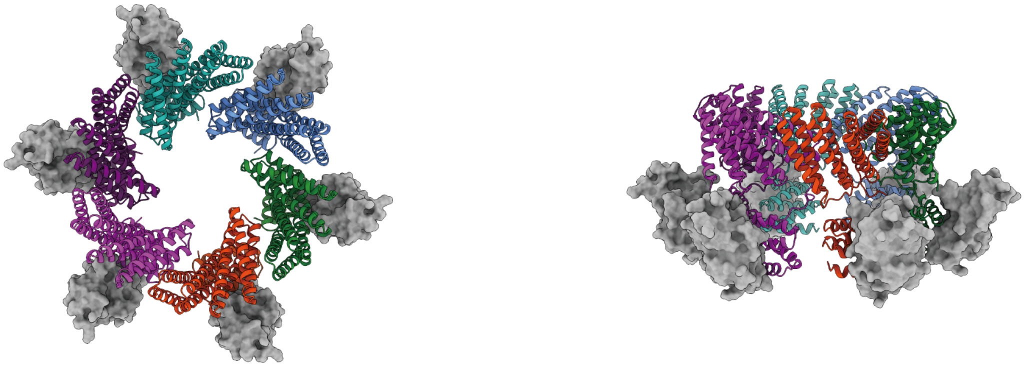

Figure 11. 5HCS_CTLA4_1 fused with hexamers. Top view (left) and side view (right) of the 5HCS_CTLA4_1 fusion with hexamers (cartoon) in complex with CTLA-4 (grey surface). Each chain is colored by a different color.

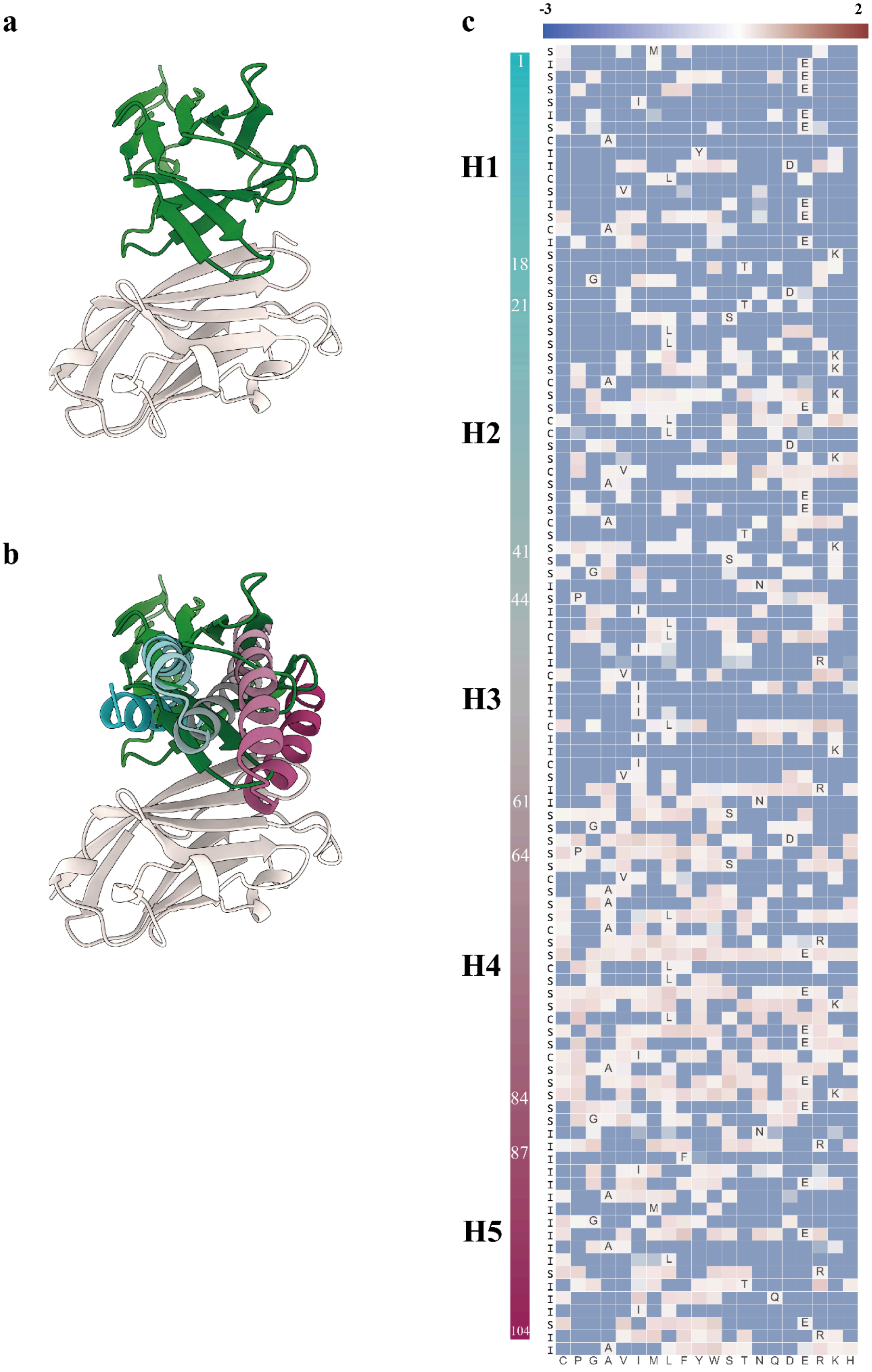
a
b
c
-3
2
H1
H2
H3
H4
H5
1
18
21
41
44
61
64
84
87
104
C P G A V I M L F Y W S T N Q D E R K H

Figure 12. PD-L1 binding protein binding site and SSM analysis. a. Complex structure of the PD-L1 (gray) and the PD-1 (green). **b.** Alignment of 5HCS_PDL1_1 (rainbow) design model to PD-L1 (gray) and binds to the PD-1 (green) binding site. **c.** Heat map representing the log enrichments[3] for the 5HCS_PDL1_1 SSM library selected with 6 nM PD-L1. Enriched mutations are shown in red and depleted in blue. The annotated amino acid in each row on the heatmap indicates the residue from the parent sequence. Residues are categorized into interface(I), core(c) and surface(S) by their position in the model. The category is listed beside the heatmap on the left.

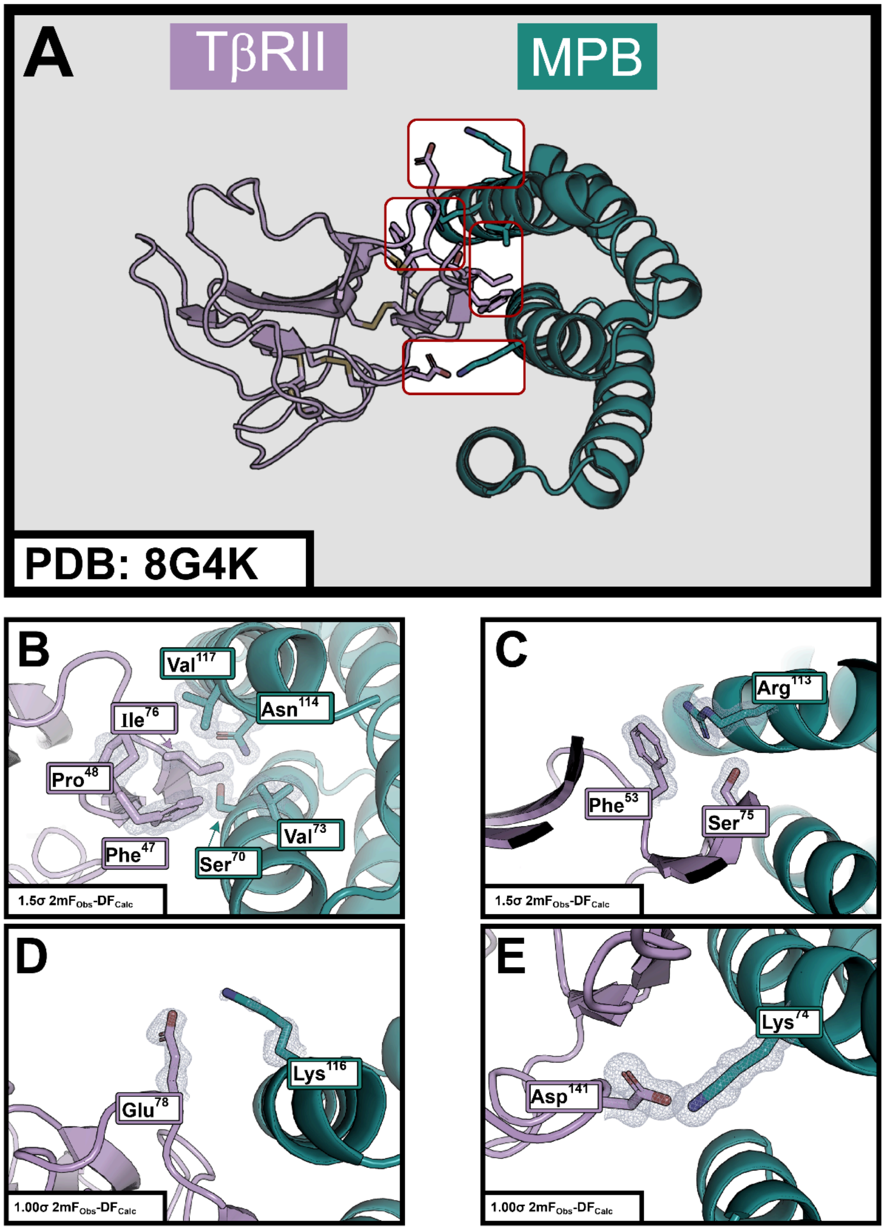

Figure 13. A portion of the electron density map for 8G4K. Representative electron density map for the 8G4K crystal structure. The map is contoured at 1.0, 1.5 σ and

corresponds to 2mFo-Fc maps. The displayed region highlights key structural features, providing insight into the quality and accuracy of the model.

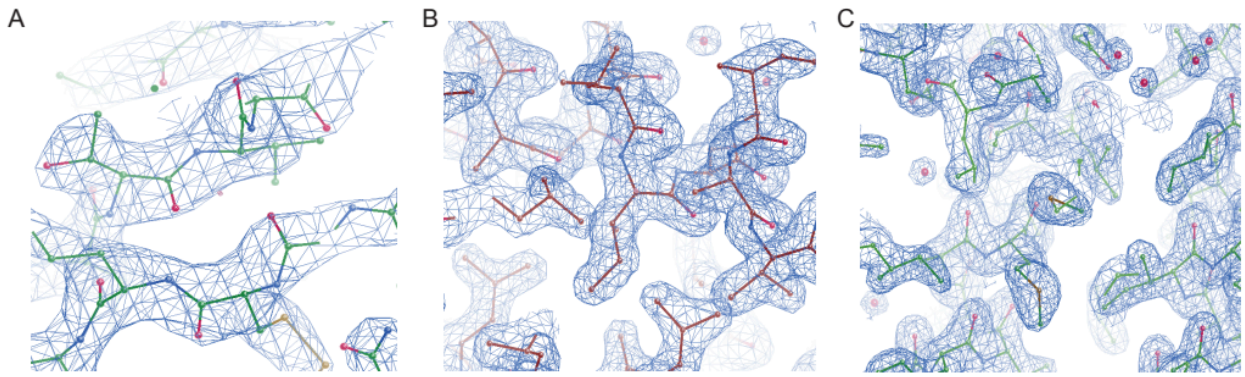

Figure 14. A portion of the electron density maps for 8GAB, 8GAC, and 8GAD. A) 2Fo -Fc maps of CTLA4 and CTLA4 binder complex (PDB ID 8GAB) contoured at 1.2 σ (blue mesh). B) 2Fo -Fc maps of CTLA4 binder (PDB ID 8GAC) contoured at 1.5 σ. C) 2Fo -Fc maps of PD-L1 binder (PDB ID 8GAD) contoured at 1.5 σ.

Table 1: Crystallization conditions and Crystallographic data statistics

**Protein**	**TGFβRII binder (5HCS_TGBR2_1) complex**	**CTLA-4 binder (5HCS_CTLA4_1) complex**	**CTLA-4 binder (5HCS_CTLA4_2)**	**PD-L1 binder (5HCS_PDL1_1)**
**Ligand**[$]	**none**	**none**	**none**	**Indole**
**Crystallization condition**	20% (w/v) PEG-MME 5K, 0.4 M $(NH_4)_2$ SO_4, 0.1 M Tris pH 7.4, and 16 – 32 % glycerol	22% (w/v) PEG 3350 and 0.2 M KCl	30 % (w/v) PEG 3350, 0.22 M $MgCl_2$ and 0.0.8 M Bis-Tris pH 5.5 and 0.15 M HEPES pH 7.5	22.5% (w/v) PEG 3350, 0.08 M Bis-Tris pH 5.5 and 2% (v/v) Ethylene glycol
**Cryo preservation**	16 - 32% glycerol	8% (v/v) Ethylene glycol	5% (v/v) Ethylene glycol	8% (v/v) Ethylene glycol
**Data Collection***				
Source	APS 22-ID	BNL 17-ID-2	APS 31-ID	BNL 17-ID-1
Wavelength (Å)	1.00	0.98	0.98	0.92
Number of crystals	1	1	1	1
Space group	$P2_12_12_1$	C2	$P2_12_12_1$	$P4_32_12$
Cell dimensions				
a,b,c (Å)	47.98, 57.17, 78.80	175.75, 33.6,74.47; β = 101.15	39.9, 52.1, 53.5	67.24, 67.24, 101.9
Resolution (Å)	46.28-1.24(1.27-1.24)	20-2.72 (2.82-2.72)	20-1.85 (1.92-1.85)	20-1.88 (1.95-1.88)
Completeness (%)	95.7(53.4)	99.8 (99.3)	99.6 (99.9)	99.8 (99.3)
Total reflections	547697	44220	105488	286708
Unique reflections	61539	11876	9930	19508
Wilson B-factor	14.1	55.07	26.4	36.85
Multiplicity	8.9(2.3)	3.7 (3.6)	10.6 (10.9)	14.7 (14.9)
R_{merge} (%)	5.2(89.8)	8.7 (89.7)	8 (80.3)	5.9 (84.7)

$CC_{1/2}$ (%)	100(42.5)	99.9 (87.2)	99.9 (84.4)	99.9 (89.1)
CC* (%)	100(77.2)	99.6 (87.6)	100 (96.9)	100 (97.1)
<I>/s(I)	18.9(0.6)	10.35 (2.08)	18.4 (3.4)	30.24 (3.66)
**Refinement***				
Resolution (Å)	46.28-1.24	19.5-2.72 (2.82 - 2.72)	19.9-1.85 (2.12-1.85)	19.62-1.88 (1.95-1.88)
Reflections: work/free	61539/3078	11858 (1170)/548 (67)	9928 (985)/430 (45)	19506 (1888)/983 (90)
R_{work}/R_{free} (%)	18.13/19.58	28.7 (32.6)/32.9 (37.4)	19.1 (23.4)/21.8 (26.7)	18.9 (25.7)/21.2 (29.9)
Number of TLS groups	0	0	4	8
Number of atoms				
Protein	3517	3482	2200	1632
Ligand	5	0	4	9
Water	201	0	63	103
Average B-factors ($Å^2$)				
Protein	20.92	50.3	29.1	43.23
Ligand	30.0		39.6	51.75
Water	34.96		38.9	49.93
rmsd.				
Bond lengths (Å)	0.0198	0.004	0.01	0.007
Bond angles (°)	1.9599	0.73	1.1	0.88
MolProbity†				
Favored	98.6% (217 aa)	98% (394 aa)	100% (98 aa)	99.5% (208 aa)
Allowed	100% (220 aa)	100% (402 aa)	none	100% (209 aa)
Outliers	none	none	none	none
Clash score	100th percentile	98th percentile	100th percentile	100th percentile

Molprobity score	100th percentile	98th percentile	100th percentile	100th percentile
**RCSB ID**	8G4K	8GAB	8GAC	8GAD

* Statistics calculated using Phenix (Adam et al., 2010); highest resolution shells indicated in parentheses

† Calculated with the program MolProbity (Chen et al., 2010)

Table 2. Sequences in this study.

ID	Sequence
5HCS_TGFBR2_0	GLKELLKELEKAIASGDTETVRRILEELLELLKVAFEKGDYAEAISIAALAVKAAAYLGDTETLKELLEI<br>LKKIKEKLKKIGDETLLKAVERNIKVVEKLA
5HCS_TGFBR2_1	GLKELLKELNKAIASGDTETVRRILEELLELLKEAFEKGDYDLAISIASMAVKAASYIGDTETLKELLEI<br>LKKIKEKLKKEGDEAALKAVERNIKVVEKVA
5HCS_CTLA4_0	NNLEEVVKTLLKELSSIFFLQNHEASHEALHRVLEVLKLLLEHNNLELATTLISIAVHVAVRVNDEHVI<br>RELRHLIRRLLKLIKEHNDNQLFVAVVSVIMYLERY
5HCS_CTLA4_1	NNLEEEVHKLLWELSEIYHHHDHEASHEALRRALEVLKQLLEHNNLEQAVTLVSIAVHVAVRVNDEH<br>VIRELRHFLRRLLKQVKEHNNNKLALLVMSVKMQLDRT
5HCS_CTLA4_2	NNLEEEVQKLLYELSEIWHQHDHEASREALHRVLEVLKQLLEHNNLEQAVELISIAVHVAVRVNNEH<br>VIRELHHLLRRLLKQVKEHNNNKLYIAVMSVIMQLERT
5HCS_CTLA4_1_c6	NNLEEEVHKLLWELSEIYHHHDHEASHEALRRALEVLKQLLEHNNLEQAVTLVSIAVHVAVRVNDEH<br>VIRELRHFLRRLLKQVKEHNNNKLALLVMSVKMQLDRTGGSGGSGGSSSDEEEARELEERAREA<br>AKRAIEAAKRTGDPRVRELAEELVKLAIWAAVEVWLDPSSSDVNEALKLIVEAIEAAVRALEAAERT<br>GDPEVRELARELVRLAVEAAEEVQRNPSSSDVNEALKLIVIAIEAAVRALEAAERTGDPEVRELARE<br>LVRLAVEAAEEVQRNPSSEEVNEALRKIIKLILFAVMVLELAEEIGDPTWREMARRAVREAVELAEE<br>VQRDPSGWLGH
5HCS_PDL1_0	MEEEIDEAYDLVEEAEKTGDTSLLKKAKELLDKVAEEAVKSGNPKLLIRVVILLLKIVRLLGDPSVAAL<br>ARELLEKLEEIAEKEGNEYIEAFGEALRRDIERVL
5HCS_PDL1_1	MEEEIEEAYDLVEEAEKTGDTSLLKKAKELLDKVAEEATKSGNPILLIRVIIILIKIVRNSGDPSVAALAR<br>ELLEKLEEIAEKEGNRFIEAMGEALRTQIERAL

Reference

1. Jumper, J. *et al.* Highly accurate protein structure prediction with AlphaFold. *Nature* **596**, 583–589 (2021).
2. Hiranuma, N. *et al.* Improved protein structure refinement guided by deep learning based accuracy estimation. *Nat. Commun.* **12**, 1340 (2021).
3. Bryan, C. M. *et al.* Computational design of a synthetic PD-1 agonist. *Proc. Natl. Acad. Sci.* **118**, e2102164118 (2021).
